# Challenges in Chlamydial Serology: Insights from a Belgian and a Dutch Population Cohort

**DOI:** 10.3390/microorganisms12040658

**Published:** 2024-03-26

**Authors:** Anne De Meyst, Zoïe Alexiou, Tinne Lernout, Servaas A. Morré, Daisy Vanrompay

**Affiliations:** 1 Laboratory of Immunology and Animal Biotechnology, Department of Animal Sciences and Aquatic Ecology, Faculty of Bioscience Engineering, Ghent University, 9000 Ghent, Belgium; anne.demeyst@ugent.be; 2 Centre for Infectious Disease Control, National Institute for Public Health and the Environment, P.O. Box 1, 3720 BA Bilthoven, The Netherlands; zoie.alexiou@rivm.nl; 3 Institute for Public Health Genomics (IPHG), GROW Research Institute for Oncology and Reproduction, Maastricht University, 6211 LK Maastricht, The Netherlands; samorretravel@yahoo.co.uk; 4 Epidemiology of Infectious Diseases, Epidemiology and Public Health, Sciensano, 1050 Brussels, Belgium; tinne.lernout@sciensano.be; 5 Dutch *Chlamydia trachomatis* Reference Laboratory, Deptartment Medical Microbiology, Faculty of Health, Medicine & Life Sciences, Maastricht University, 6229 ER Maastricht, The Netherlands; 6 Department of Molecular and Cellular Engineering, Jacob Institute of Biotechnology and Bioengineering, Sam Higginbottom University of Agriculture, Technology and Sciences, Allahabad 211007, Uttar Pradesh, India

**Keywords:** serology, *Chlamydia*, diagnostics, *C. trachomatis*, *C. pneumoniae*, *C. psittaci*

## Abstract

Serology routinely serves as a diagnostic tool to confirm *Chlamydia* infections in humans. Particularly in delayed settings, such as post-outbreak scenarios where the acute phase of infection has subsided, serology is invaluable. Multiple studies, nonetheless, indicate deficiencies in specificity and sensitivity of current chlamydial antibody detection assays. Incorporation of multiple antigens per target is known to improve the accuracy of chlamydial serological assays. We, therefore, used the recomLine test (Mikrogen diagnostics) on serological samples of two cohorts, as it is the only commercially available test allowing detection of antibodies against three human pathogenic *Chlamydia* species (*C. trachomatis*, *C. pneumoniae* and *C. psittaci*) using multiple antigens per target. The first cohort (n = 156; samples collected between 2008 and 2022 during a *C. trachomatis* screening initiative) comprised women from the Netherlands (NL) with past exposure to *C. trachomatis*, while the second cohort (n = 44; samples collected in 2018 in a health examination survey) consisted of Belgian citizens (BE) with occupational or recreational exposure to chickens, representing a risk population for *C. psittaci*. The test indicated a statistically equivalent *C. pneumoniae* seroprevalence in both cohorts (39.10% in NL and 34.09% in BE; p = 0.337). As expected *C. trachomatis* seroprevalence was significantly higher (p < 0.001) in the Dutch cohort (48.72%), as compared to the Belgian cohort (4.55%). Lastly, *C. psittaci* seroprevalence did not significantly differ between the two groups (2.27% in BE and 1.92% in NL; p = 0.633), even though a higher prevalence was expected for the Belgian cohort. This prompts us to question whether the Belgian cohort truly constituted a *C. psittaci* risk population or whether the recomLine test is susceptible to cross-reaction of species-specific antibodies, thereby increasing *C. psittaci* prevalence in the Dutch cohort. We advocate for the development of affordable, highly sensitive antibody detection assays that can effectively distinguish between chlamydial species, addressing the increasing demand for enhanced serological testing methodologies.

## 1. Introduction

Worldwide, bacteria from the genus *Chlamydia* (C.) are infecting animals and humans. These obligate intracellular Gram-negative bacteria consist of 14 species and 4 candidatus species, from which 3 species, namely *C. trachomatis*, *C. pneumoniae* and *C. psittaci*, are regularly detected in humans [1].

*C. trachomatis*, the best known human pathogen of the genus, is considered the most clinically important. Serovars A–C cause ocular infections which might result in chronic inflammation of the eyelids [2]. *C. trachomatis* is also responsible for the most common bacterial Sexually Transmitted Disease (STD). Serovars D–K and L1–L3 infections are in 80% of the cases asymptomatic, but if left untreated, they can cause pelvic inflammatory disease leading to subfertility in women, ectopic pregnancy or lymphogranuloma venereum [3,4].

*C. pneumoniae*, the second species affecting humans, was initially considered to be associated with acute human respiratory disease. While most infections are asymptomatic or mild, the incidence of *C. pneumoniae* in community-acquired pneumonia (CAP) is still estimated to be around 10%. *C. pneumoniae* has also been found in a variety of animals like horses, koalas and frogs [5,6].

The last *Chlamydia* species that is frequently detected in humans is *C. psittaci*. While it is primarily an avian pathogen, it can be transmitted to humans, causing psittacosis—an internationally notifiable disease. Infections manifest as flu-like illness, including fever, headaches and pneumonia [7]. *C. psittaci* is responsible for approximately 1% of all CAP cases in hospitalized patients worldwide [8]. Case confirmation in Belgium requires isolation of *C. psittaci* from respiratory secretions or blood, or a significant rise in chlamydial serum antibodies. In the Netherlands, a positive result from a *Chlamydia*-specific Nucleic Acid Amplification Technique (NAAT) also suffices. After notification, contact tracing and cluster detection are initiated to prevent further zoonotic events [9,10].

Presently, NAATs are recognized as the gold standard for *Chlamydia* diagnostics, owing to their high sensitivity and specificity. However, as NAATs only provide information on a single point of time, chronic or past infections are preferably diagnosed with antibody detection assays. Additionally, these assays can be used to estimate population-level exposure, to provide insight on infection age or nature (acute or chronic), or for official case confirmation in case of a suspected psittacosis diagnosis [10,11,12,13]. Existing commercial chlamydial serological assays, primarily consisting of Enzyme-Linked Immunosorbent assays (ELISAs) or Micro-Immunofluorescence assays (MIFs), often lack the required sensitivity and specificity for effective use in epidemiological research and diagnosis. *Chlamydia*-specific ELISAs, characterized by their high-throughput nature, may encounter decreased species-specificity due to the use of inactivated whole-organisms, chlamydia lysates or (recombinant) antigens containing sequence-conserved regions (e.g., the Major Outer Membrane Protein; MOMP), and thus, inevitable cross-reactivity of genus-specific antibodies. MIF is regarded as the serological gold standard for *C. trachomatis* and has been adapted for detection of *C. pneumoniae-* and *C. psittaci*-antibodies because of its higher sensitivity compared to ELISA [14]. Despite its susceptibility to cross-reaction, inherent to the use of whole-organisms or sequence-conserved antigens, MIF allows skilled personnel to identify a pattern of specific versus non-specific signal during microscopic evaluation, improving specificity. Especially, the detection of *C. trachomatis* anti-MOMP antibodies occurs with high sensitivity and specificity, but for *C. pneumoniae* this is less the case due to the lower immunogenicity of its MOMP protein [14,15]. Overall, MIF remains a cumbersome procedure, prone to high inter-laboratory variation and still often lacks sensitivity and specificity. Consequently, there is a clear need for improved serological assays for use as a diagnostic and epidemiological tool [14,16]. In general, the use of immunoassays relying on whole organisms or cell lysate is strongly discouraged. The use of a single species-specific antigen decreases sensitivity due to the stochastic nature of the antibody response and is, therefore, also inadvisable. The complexity of serology on human *Chlamydia* pathogens is further increased by the occurrence of mixed chlamydial infections in the same host, necessitating an assay capable of accurately distinguishing between the three species. To address these challenges, researchers have identified highly reactive and specific B cell epitopes for each species for use in chlamydial serology. The combination of multiple species-specific epitopes has been shown to improve both sensitivity and specificity and is currently considered the most optimal strategy for chlamydial serology [14,16,17,18].

This study used a commercial serological assay that incorporates multiple species-specific antigens, yielding elevated sensitivity and specificity. The assay is an immunoblot which examines the presence of species-specific antibodies against *C. trachomatis-*, *C. pneumoniae*- and *C. psittaci*-specific antigens. Prevalence rates of the three human *Chlamydia* pathogens were compared between two cohorts: a Dutch cohort consisting of women with prior exposure to *C. trachomatis* and a Belgian cohort comprising individuals with regular contact with chickens, representing a risk group for *C. psittaci* infections. With this study, we aimed to test the use of an existing *Chlamydia* species-specific serological assay (the recomLine *Chlamydia* IgG kit (Mikrogen Diagnostik, Neuried, Germany)) to gain insight into *Chlamydia* epidemiology in two diverse cohorts.

## 2. Materials and Methods

### 2.1. Collection of Samples

In the Netherlands, women of reproductive age were recruited for a population-based *C. trachomatis* screening initiative starting in 2008 and followed for up to 14 years [19]. During that initiative, participants provided questionnaire data, blood samples and host DNA to determine specific genetic biomarkers related to susceptibility and severity of infection. The recruited women all resided in urban areas of Amsterdam and Rotterdam, as well as in a defined suburban area of south Limburg (Parkstad), at the start of the screening program. Past exposure to *C. trachomatis* was estimated by *C. trachomatis* PCR testing, self-reported *C. trachomatis* diagnosis and/or testing for *C. trachomatis* IgG antibodies. Only a subset of women with confirmed past exposure to *C. trachomatis* and with sufficient available serum were selected for inclusion in this study (n = 156).

In Belgium, a Health Examination Survey (BELHES) was conducted in 2018, involving 1.184 Belgian residents aged 18 years and older [20]. During that survey, participants underwent a clinical examination and provided questionnaire data and blood and urine samples. This study specifically involved a subgroup of participants who provided additional informed consent, had verified exposure to chickens (professionally or recreationally) at the time of sampling, and provided a serum sample (n = 44).

### 2.2. Serology

The two hundred sera were analysed with the recomLine *Chlamydia* IgG (Mikrogen Diagnostik, Neuried, Germany), executed by one and the same person to avoid interlaboratory variation. The test principle allows the identification of specific antibodies against various antigens of *C. trachomatis* (MOMP, OMP2, TARP, CPAF, HSP60), *C. pneumoniae* (MOMP, OMP2, TARP, CPAF, YwbM) and *C. psittaci* (MOMP, OMP2, TARP, CPAF), through the separate line-up of the individual antigens. The test was executed and interpreted according to manufacturer’s guidelines. Briefly, the blot was run and points were assigned to *Chlamydia* antigens whose band intensity exceeded the cut-off level, as shown in Table 1. Subsequently, points were added up per sample and samples were classified as negative, borderline, or positive, following the criteria outlined in Table 2. According to the manufacturers, the diagnostic sensitivity of the kit was 100%, 99% and 100% for *C. trachomatis*, *C. pneumoniae* and *C. psittaci*, respectively, including borderline results. Test specificity for all three species was 100%.

### 2.3. Statistical Analysis

Species-specific prevalence rates were statistically compared between cohorts using a Chi-square test in SPSS Statistics 28, with a significance level of 0.05.

## 3. Results

The Dutch cohort included 156 women with a median age of 35 years. The Belgian cohort consisted of 24 men and 20 women with a median age class between 45 and 54 years. Samples were defined as seropositive, borderline or negative, according to manufacturer’s guidelines. In Table 3 and Table 4, the prevalence rates are presented, respectively including and excluding borderline cases to the *Chlamydia*-positive samples. *C. trachomatis* prevalence significantly differed between both cohorts, with a prevalence of 4.55% in the Belgian cohort and a prevalence of 51.92% in the Dutch cohort (Table 3). *C. pneumoniae* and *C. psittaci* prevalences were not significantly different between cohorts. The *C. pneumoniae* prevalence in the Belgian and Dutch cohort was respectively equal to 36.36% and 42.31%, whereas for *C. psittaci* the prevalence was equal to 4.55% and 10.26%, respectively (Table 3). Overall, prevalence rates remained in a similar range when excluding borderline cases, except for *C. psittaci* in the Dutch cohort (Table 4). *C. psittaci* prevalence in that cohort decreased from ~10% to ~2%, as 13/140 samples were considered borderline samples and only 3/140 were clearly positive, according to the manufacturer’s case definition.

## 4. Discussion

Immunoblot analysis was conducted on sera from two different cohorts, to examine the presence of antibodies targeting membrane proteins, inclusion membrane proteins, and intracellular proteins of *C. trachomatis*, *C. pneumoniae*, and *C. psittaci*. The utilized assay is currently the only commercially available method capable of detecting multiple species-specific antigens, targeting the three human *Chlamydia* pathogens in a single assay. The manufacturers supplied a manual for result interpretation, enabling the classification of samples into *Chlamydia* IgG positive, borderline, or negative samples. Unfortunately, the manual did not specify whether borderline cases should be considered positive or negative, and additional information on the rationale behind the classification system was lacking as well. Therefore, in this study, we once included and once excluded borderline cases of the positive samples. Both approaches yielded similar prevalence rates for the three *Chlamydia* species, except for *C. psittaci* in the Dutch cohort. Upon closer examination, it appeared that 13/140 samples from that cohort were classified as *C. psittaci* borderline cases and only 3/140 samples were judged as *C. psittaci*-positive. Notably, all 13 borderline cases were also positive for *C. trachomatis*, whereas only 1 out of 3 *C. psittaci*-positive samples was also positive for *C. trachomatis*. These observations suggest a degree of cross-reaction and indicate that the classification system might have been designed to address this phenomenon. Consequently, prevalence rates which excluded borderline cases were deemed more accurate.

The prevalence of *C. trachomatis* was significantly higher in the Dutch cohort compared to the Belgian cohort (Table 4). This discrepancy was expected, given that the Dutch cohort consisted of women with a prior exposure to *C. trachomatis*. In the general population of the Netherlands, IgG seroprevalence typically ranges between 6 and 11%, depending on factors such as gender and age, aligning well with the results observed in the Belgian cohort [21]. Additionally, literature indicated that 41.6–60.8% of the *C. trachomatis* NAAT-positive people produce antibodies detectable with current commercial assays, explaining the prevalence of around 50% in the Dutch cohort [22,23]. Indeed, a *Chlamydia* infection does not automatically trigger the production of antibodies. In some individuals, genetic variations in pattern recognition receptors can lead to inadequate recognition of *C. trachomatis*, increasing susceptibility to persistence [24,25].

*C. pneumoniae* prevalence in the Belgian and Dutch cohort was equal to 34.09% and 39.10%, respectively, with no significant difference between the two cohorts. Earlier studies reported varying prevalence rates in asymptomatic adults, ranging from 58.9% in Jordanian adults [26] and 64.9% in German adults [27], to 77.7% in Finnish adults [28]. Generally, *C. pneumoniae* seroprevalence increases with age and is notably influenced by the employed testing method [29,30,31].

Lastly, the prevalence of *C. psittaci* showed no significant difference between the two cohorts (NL: 1.92% versus BE: 2.27%). Prevalence rates were expected to be higher in the Belgian cohort as it comprised individuals who were exposed to chickens at the time of sampling, previously-defined as a risk population [32]. However, since there was no information collected on the frequency or duration of exposure, it is not possible to assess if the level of exposure might have been too low, but it could be a hypothetical explanation for the observed similar prevalence rates between the Dutch and Belgian cohorts. Another possible explanation is the potential overestimation of *C. psittaci* prevalence in the Dutch cohort due to cross-reaction between *C. trachomatis* antibodies and *C. psittaci* antigens. When accounting borderline cases as positive samples, this indeed seems to be plausible, but as the prevalence rates between both cohorts were still similar after the exclusion of borderline cases, this hypothesis is less likely. A third explanation could be the lack of antibody production when infected. Similar to *C. trachomatis*, the absence of a humoral antibody response has been reported in hospitalized patients with a confirmed diagnosis of psittacosis [33,34]. Whether the low incidence of *C. psittaci* in the Belgian cohort was indeed a result of limited exposure or a consequence of the absence of an antibody response, will remain unknown. Nevertheless, this finding confirms our opinion that serology should not be used for diagnostic purposes (including official case confirmation), possibly missing an infection due to the absence of a humoral immune response.

While the recomLine test is suboptimal for diagnostic purposes, it did offer valuable insights into *Chlamydia* exposure levels in two distinct cohorts. This enhanced serological assay is, therefore, ideal for use in epidemiological research and outbreak scenarios. However, we do recommend the use of *Chlamydia* species-specific NAATs, with or without species-specific antibody detection, for a comprehensive and accurate diagnosis of *Chlamydia* infections in humans. Additionally, in settings where adequate facilities or trained personnel are lacking for NAAT diagnosis, point-of-care (POC) tests may offer a valuable solution. Unfortunately, existing chlamydial POC tests, mostly targeting *C. trachomatis*, lack the required sensitivity and specificity [35,36]. The development and availability of improved POC tests, alongside enhanced standard diagnostic methods, would enhance *Chlamydia* diagnosing, contributing to the reduction of the global health burden.

In summary, we advocate for increased accessibility to sensitive and specific antibody detection assays, like the recomLine test, especially for use in epidemiological research and outbreak scenarios. We further emphasize the importance of transparent communication among manufacturers, researchers, and clinicians to facilitate accurate results interpretation. Lastly, we advise against the exclusive use of *Chlamydia* antibody detection assays and recommend *Chlamydia* species-specific NAATs in *Chlamydia* diagnostics.

## Figures and Tables

**Table 1:** Point assessment of *Chlamydia* antigens. (*) 6 points if the OMP2 of *C. trachomatis* and *C. psittaci* are negative, otherwise 2 points. (**) 6 points if the MOMP of *C. trachomatis* and of *C. pneumoniae* are negative, otherwise 4 points.

Antigen	*C. trachomatis* Antigens	*C. pneumoniae* Antigens	*C. psittaci* Antigens
MOMP	6	6	4/6 **
OMP2	2	2/6 *	1
TARP	3	3	3
CPAF	3	3	3
HSP60	1	-	-
YwbM	-	6	-

**Table 2:** Interpretation of the results in recomLine *Chlamydia*.

Points’ Total	Assessment
≤3	negative
4–5	borderline
≥6	positive

**Table 3:** Seroprevalence rates determined with recomLine *Chlamydia* IgG, including borderline cases to *Chlamydia*-positive samples. Results are presented as “prevalence rate in % (95% confidence interval in %)”.

	*C. trachomatis*	*C. pneumoniae*	*C. psittaci*
Belgian cohort (n = 44)	4.55% (1.41–7.69%)	36.36% (29.11–43.62%)	4.55% (1.41–7.69%)
Dutch cohort (n = 156)	51.92% (47.92–55.92%)	42.31% (38.35–46.26%)	10.26% (7.83–12.69%)
Significance	p < 0.001	p = 0.298	p = 0.196

**Table 4:** Seroprevalence rates determined with recomLine *Chlamydia* IgG, excluding borderline cases to *Chlamydia*-positive samples. Results are presented as “prevalence rate in % (95% confidence interval in %)”.

	*C. trachomatis*	*C. pneumoniae*	*C. psittaci*
Belgian cohort (n = 44)	4.55% (1.41–7.69%)	34.09% (26.94–41.24%)	2.27% (0.03–4.52%)
Dutch cohort (n = 156)	48.72% (44.72–52.72%)	39.10% (35.20–43.01%)	1.92% (0.82–3.02%)
Significance	p < 0.001	p = 0.337	p = 0.633

## Data Availability

Data are contained within the article.
